# Advances in the beneficial effects of nutrition on stroke-related Sarcopenia: A narrative review

**DOI:** 10.1097/MD.0000000000034048

**Published:** 2023-06-16

**Authors:** Zhiqiang Gao, Hongxia Chen

**Affiliations:** a School of Public Health, Hubei University of Medicine, Shiyan, China; b Institute of Biomedical Research, Taihe Hospital, Hubei University of Medicine, Shiyan, China.

**Keywords:** assessment, geriatric nutritional risk index, malnutrition, nutritional supplementation, sarcopenia, stroke

## Abstract

Stroke is one of the most common causes of disability in adults. Sarcopenia is a syndrome characterized by progressive systemic muscle loss and functional decline. The decrease in skeletal muscle mass and muscle function throughout the body after stroke cannot be explained by neurological motor disorders due to brain injury alone, it is considered to be a secondary sarcopenia known as stroke-related sarcopenia. Mounting evidences showed that stroke-related sarcopenia might promote the occurrence and development of sarcopenia through various pathogenesis such as muscle atrophy, dysphagia, inflammation, and malnutrition, etc. At present, the main indicators used to assess malnutrition in patients with stroke-related sarcopenia include temporalis muscle thickness, calf circumference, phase angle, geriatric nutritional risk index and mini-nutritional assessment short-form, etc. Currently, there is no particularly effective method to curb its progression, but supplementation with essential amino acids, whey protein combined with vitamin D, high energy diet, avoiding Polypharmacy, as well as increasing physical activity level and reducing sedentary lifestyle may improve the malnutrition status of stroke patients, and increase the muscle mass and skeletal muscle index, further delay or even prevent the development of stroke-related sarcopenia. This article reviews the latest research progress on the characteristics, epidemiology, pathogenesis and the role of nutrition in stroke-related sarcopenia, so as to provide reference for the clinical treatment and rehabilitation of stroke-related sarcopenia.

## 1. Introduction

Sarcopenia is a syndrome characterized by progressive decrease in skeletal muscle mass associated with aging, accompanied by decline in muscle strength and/or muscle function, and can cause a series of adverse consequences.^[1]^ Clinically, without clear boundaries, sarcopenia is divided into primary sarcopenia (age-related) and secondary sarcopenia (activity related, disease related, and nutrition related).^[2]^ Stroke is the leading cause of adult disability and the second cause of death worldwide. “Stroked-related” sarcopenia is regarded as the result of progressive muscle atrophy induced by neurological disorders, inflammation, and inactivity,^[3]^ which has distinctive characteristics, such as rapid loss of muscle mass, structural changes of muscle (shift of muscle fibers to fast twitch fibers), differential performance of bilateral limb function due to brain damage, muscle atrophy independent of age, and activation of catabolic signals with neurotrophic imbalance.

The prevalence of stroke-related sarcopenia ranges from 14% to 54%, depending on geographic region, diagnostic criteria and time of onset of stroke patients included in the study.^[4]^ Studies^[5]^ have found that the prevalence of pre - and post-stroke sarcopenia increases over time, increasing by about 15% from pre-stroke to 10 days after stroke, and by about 20% from 10 days to 1 month after stroke. A meta-analysis found that the prevalence of stroke-related sarcopenia was 42%.^[6]^ Specifically, the prevalence of sarcopenia is 14% to 18% in community stroke population and 48.3% to 60.3% in rehabilitation ward.^[7]^The prevalence of stroke is expected to increase in the future, with a concomitant increase in the prevalence of stroke-related sarcopenia.

Muscle atrophy is a major cause of post-stroke frailty, and the decrease in skeletal muscle mass and muscle function cannot be explained by neurological damage induced motor disorders due to brain injury alone. The exact mechanism of stroke-associated sarcopenia is not clear yet, and may be associated with a combination of mechanisms such as muscle atrophy and apraxia, dysphagia, inflammatory response and malnutrition.

Muscle denervation and inability to perform physical activity due to loss of cortical control after stroke increases the risk of muscle dystrophy. Muscle atrophy is a common complication of stroke, and muscle damage after stroke can cause decreased physical function or disability. At the same time, skeletal muscle injury leads to motor unit degeneration, paralysis, and immobility, accompanied by skeletal muscle atrophy. Skeletal muscle protein synthesis and degradation in healthy adults is a balanced and dynamic process.^[6]^ Progressive loss of muscle tissue and decline in muscle mass and strength may occur during normal aging. However, in the case of accelerated energy and muscle wasting during the disease, the process of stroke-related sarcopenia will be accelerated. It has been found that the number of motor units in muscle tissue begins to decrease 4 hours after cerebral infarction, which is because of the transsynaptic inhibition of spinal α motor neurons involved in the supply of the muscle.^[8]^ Decreased physical activity level is the main cause of sarcopenia. Studies have found that patients with acute stroke exercise for less than 40 minutes per day during hospitalization, long-term bed rest leads to a decline in muscle strength earlier than the loss of muscle mass. Low-intensity activity causes muscle strength decline, muscle weakness further reduces exercise capacity, which forms a vicious circle. In addition, since stroke patients usually have a sedentary lifestyle prior to stroke, the risk of sarcopenia is obviously increased.

Dysphagia is one of the major complications after stroke. Swallowing involves multiple muscles and nerves that depend on the central nervous system for sensory feedback, motor programming and execution, and processing.^[9]^ Stroke is the most common disease that causes disruption of the swallowing network and thus dysphagia.^[10]^ Neurogenic dysphagia can be induced by nerve injury after stroke, and about 24.3% to 52.6% of stroke patients will develop dysphagia.^[11]^Therefore, oral dysphagia may lead to ineffective swallowing, causing dehydration and malnutrition, speeding up the loss of muscle mass, and affecting the rehabilitation of patients.^[12]^ Damaged brain tissue after stroke may result in impaired sensory and motor mechanisms of swallowing. Studies have found that stroke patients with dysphagia have a 2.6-fold increased risk of malnutrition.^[13]^ Catabolic pathways in muscle tissue are activated after stroke, leading to a reduction in skeletal muscle mass that may affect not only the surrounding skeletal muscle but also the muscles associated with swallowing. Severe loss of skeletal muscle mass can lead to or aggravate dysphagia, which in turn is one of the factors leading to reduced food intake in stroke patients. On the contrary, decreased food intake leads to malnutrition and loss of skeletal muscle mass, forming a vicious circle.

Systemic inflammation is closely linked to sarcopenia and poor functional outcome in stroke recovery.^[14]^ Inflammation is a major factor in the pathology and prognosis of acute ischemic stroke. Furthermore, inflammation can induce secondary brain injury by aggravating blood-brain barrier damage, microvascular failure, brain edema, oxidative stress, and direct induction of neuronal cell death.^[15]^ Stroke elicits an immune-inflammatory response characterized by strong activation of microglia, astrocytes, and vascular endothelial cells. Inflammatory cytokines can induce tissue degeneration and accelerate weight loss. Among them, the pro-inflammatory Tumor Necrosis Factor-α plays an important role in muscle mass loss. It can reduce the synthesis of protein, fat and glycogen in myofilament and skeletal muscle by regulating transcription factors, and induce the breakdown of striated muscle cells, which is closely related to the decline of muscle mass and strength. This inflammation-driven mechanism is thought to partially explain the age-related loss of muscle mass.^[16]^ Although the presence of inflammation is closely associated with complications,^[14]^ such as decreased skeletal muscle mass, decreased muscle strength, weight loss, increased cachexia, and increased all-cause mortality, it remains a challenge to uncover the specific molecular mechanisms underlying the interaction between inflammation and muscle protein metabolism. Therefore, early detection of inflammation, especially systemic inflammation and sarcopenia, can help to provide better nutritional support to restore muscle mass and improve functional recovery after stroke.

Stroke-related sarcopenia may delay post-stroke rehabilitation, worsen functional recovery, prolong the duration of hospitalization and increase the financial burden on the family. At present, there is no effective pharmacological treatment or expert consensus on the management of stroke-related sarcopenia. Nutritional supplementation may prevent the progression of disease, enhance the ability to perform activities of daily living, improve the outcomes, reduce the disease burden and better the life quality of the patients with stroke-related sarcopenia. Therefore, this article reviews the role of nutrition in stroke related sarcopenia, providing scientific basis and references for the development of clinical guidelines for stroke-related sarcopenia.

## 2. Methods

We conducted a narrative review of observational studies to investigate the beneficial effects of nutrition on stroke-related sarcopenia patients. Cross sectional, case-control, and baseline data of longitudinal studies that examined the association of serum, skeletal muscle index, physical function, and nutrition indicators with post-stroke and/or stroke-related sarcopenia were selected.

### 2.1. Selection criteria

**Figure 1.**
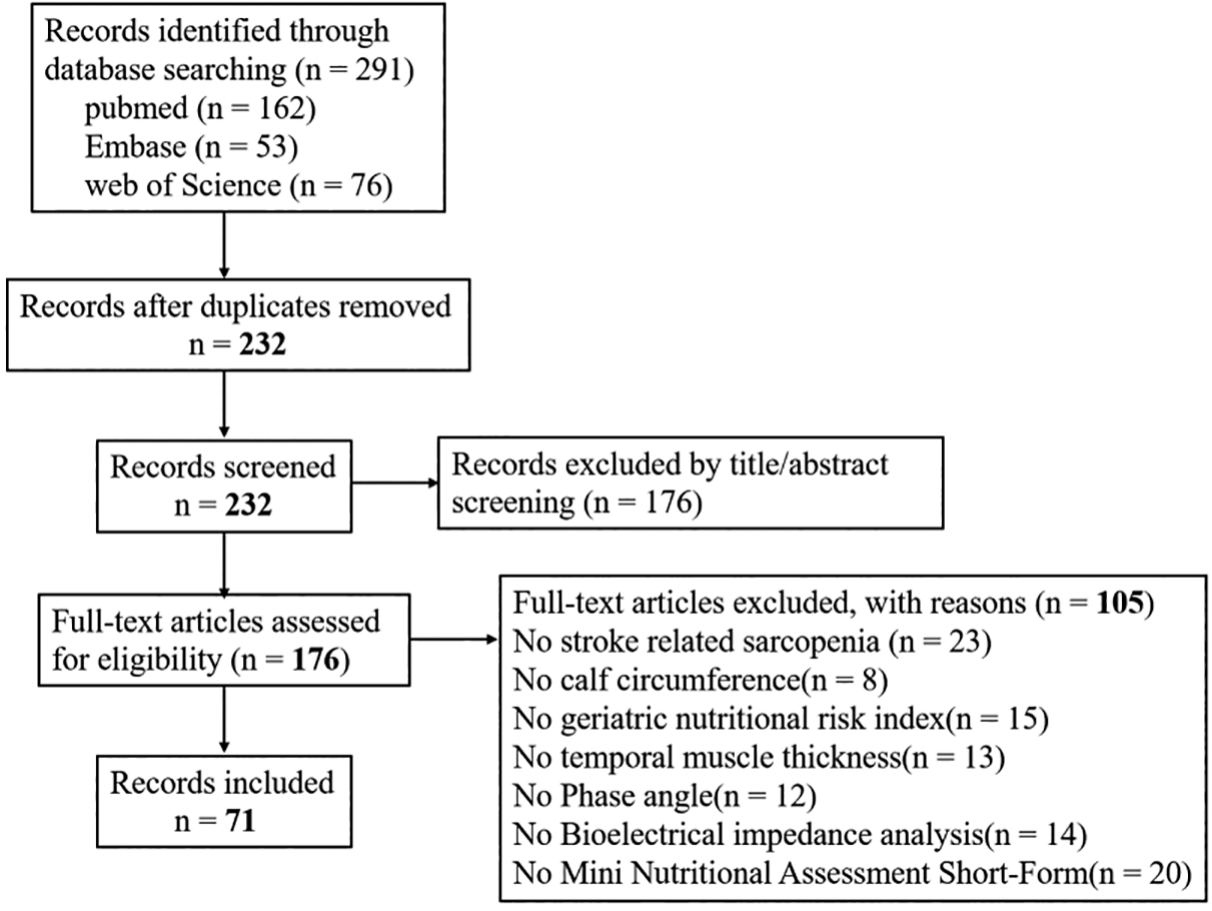
Flowchart on the search and selection of articles to be included in the review.

Inclusion criteria were: observational studies, including cross sectional, case-control, and longitudinal studies which investigated the association of supplementation nutrition on stroke-related sarcopenia with physical function in post-stroke adults as a primary or secondary outcome; participant age 60 or greater; nutrition status assessed by temporal muscle thickness, calf circumference, Phase angle, geriatric nutritional risk index, mini nutritional assessment short-form or bioelectrical impedance analysis; sarcopenia diagnosed according to the presence of muscle atrophy plus dynapenia and/or low physical function; studies published in the English language. We excluded meta-analyses, experimental, quasi-experimental, and cross-over investigations that examined intervention strategies. The selection process is shown in Figure 1.

### 2.2. Search strategy

A systematic search was undertaken in March 2023 by 2 investigators (Z.Q.G. and H.X.C.), using the following databases: PubMed, Embase, and Web of Science Core Collections. The initial search strategy was performed using keywords, MeSH terms, and free text words such as stroke, sarcopenia, nutrition.

## 3. Observations

It is well-known that malnutrition is closely related to sarcopenia. Malnutrition in patients with stroke leads to an increased risk of mortality, complications and poor functional prognosis. Early studies found that the prevalence of malnutrition in stroke patients ranges from 6.1% to 62%.^[17]^ It was reported that the prevalence of malnutrition increases over time from the onset of stroke. The meta-analysis found that the prevalence of malnutrition in acute, subacute and chronic stroke increased over time to 19%, 52%, and 72%,respectively.^[18]^ Malnutrition is common in stroke patients, and its causes are directly related to neurological diseases, such as cognitive and consciousness disorders, neurogenic vomiting, neurogenic dysphagia, depression, motor disorders and gastrointestinal dysfunction.^[19,20]^ Older adults, including those after stroke, often suffer from malnutrition, which has a negative impact on their physical function, strength and the independence of their daily life.^[21]^ Changes in nutritional status of stroke patients, such as insufficient intake and excessive nutritional consumption, will lead to a decrease in muscle synthesis and promote the occurrence of sarcopenia. Chronic malnutrition will cause the body to use skeletal muscle to provide energy, resulting in muscle loss and muscle dysfunction, leading to the onset or aggravation of myopathy. In addition, the energy and protein intake of patients after stroke will be reduced, and the lack of protein will lead to muscle nutrition loss and muscle atrophy. Therefore, adequate nutritional intake and absorption is crucially important for stroke patients, because it can promote functional recovery, brain tissue repair, prevent cognitive decline, and strengthen the immune system.

### 3.1. Indicators for assessment nutritional status of stroke-related sarcopenia

Malnutrition or inadequate nutrition intake can cause muscle decline and may lead to or aggravate post-stroke sarcopenia.^[22]^ Therefore, early nutritional status assessment is necessary in patients with stroke-related sarcopenia. At present, the main indicators used to assess the nutritional status of patients with stroke-related sarcopenia include temporal muscle thickness, calf circumference, phase angle and geriatric nutritional risk index, etc.

#### 3.1.1. Temporal muscle thickness.

Temporal muscle thickness (TMT) is a new marker for assessing muscle mass, function and nutritional status, which can be measured by computed tomography, magnetic resonance imaging and ultrasound.^[23]^ Among the older adults who were bedridden for a long time, the percentage change of TMT was significantly correlated with nutritional status, and after adjusting for age, sex and masticatory status, the percentage change of TMT was independently related to undernutrition.^[24]^ Nozoe et al^[25]^ demonstrated the reliability and effectiveness of measuring TMT as an indicator for assessing the risk of myasthenia in old adult patients with acute stroke. A total of 289 older adult patients with acute stroke were included in the study. After adjusting for potential confounding factors, multiple linear regression analysis showed that the risk of muscular reduction in older adult patients with acute stroke was independently associated with TMT. Therefore, as a novel, effective and reliable method to evaluate nutritional status, TMT is worth popularizing and applying in clinical practice.

#### 3.1.2. Calf circumference.

Currently, calf circumference (CC) is widely used as a case screening and identification tool for the diagnosis of sarcopenia, which is a vital indicator of nutritional status.^[26]^ Maeda et al^[27]^ evaluated CC as a skeletal muscle mass index and malnutrition index in 1164 hospitalized older adults. The study found that the cutoff value of CC for skeletal muscle mass and malnutrition was independently associated with hospital mortality. The cutoff value of CC is ≤26 cm in female and ≤28 cm in male, which can be used to diagnose malnutrition. Yao et al^[28]^ have found that early screening using CC measurements can easily identify stroke patients at risk of sarcopenia without the need for special equipment. Compared with other screening tools, CC has the best sensitivity, specificity, predictive value, Area Under the Curve and Kappa coefficient in screening stroke-related sarcopenia. The study by Inoue et al^[29]^ also demonstrated the effectiveness of CC in detecting stroke-related sarcopenia in older adult stroke patients. The measurement of CC is fast, low cost and easy to operate, which can be utilized as an effective indicator to quickly evaluate the nutritional status of patients with stroke-related sarcopenia, providing timely treatment and prevention for patients at risk of malnutrition.

#### 3.1.3. Phase angle.

Phase angle (PA) is an indicator derived from bioelectrical impedance technique to evaluate the nutritional status of human body, which can reflect the integrity of cell membrane and cell function as well as the nutritional status of patients. It was reported that PA is a highly predictive indicator for clinical nutritional assessment, clinical outcome and mortality of disease, with advantages of noninvasive, objective and easy to measure.^[30]^ The study had found that PA was associated with various functional indicators as well as nutritional status, weakness and sarcopenia.^[31]^ Not only with nutritional status, PA was also linked to early muscle loss after stroke. The researchers revealed the PA cutoff value between malnutrition and post-stroke sarcopenia by gender. The cutoff values for them were 5.05° for males and 3.96° for females, and 5.28° for males and 4.62° for females, respectively. Therefore, PA can be used as a indicator to distinguish malnutrition from sarcopenia in patients with acute stroke. In view of the importance of early detection and intervention of stroke-related myasthenia, PA is expected to be used as an effective parameter for initial screening of stroke-related sarcopenia.

#### 3.1.4. Geriatric nutritional risk index.

At present, Geriatric Nutritional Risk Index (GNRI)^[32]^ is mostly used as the nutritional status assessment indicator in the studies on the effects of nutrition intervention in stroke-related sarcopenia, due to the lack of expert consensus. GNRI is a biological index for evaluating nutritional status, which is calculated from the measurement of whole body albumin and ideal body weight, being a simple and accurate nutrition tool. The GNRI formula is [1.591 × serum albumin level (g/dL)] + [41.7 × current body weight (kg)/ideal body weight (kg)].^[33]^ The ideal body weight (IBW) is calculated according to the Lorentz formula calibrated for the patient’s height and sex as follows^[34]^:

for men: IBW = height (cm) − 100 − {(height (cm) − 150)/4}

for women: IBW = height (cm) − 100 − {(height (cm) − 150)/2}

According to the GNRI score Patients are categorized into: severe risk (GNRI < 82), moderate risk (82 ≤ GNRI < 92), low risk (92 ≤ GNRI < 98), and no risk (GNRI ≥ 98) of nutritional-related complications.^[35]^ GNRI has prognostic value in describing the nutritional status and nutrition-related complications of old adult hospitalized patients, and is an independent predictor of 3- and 6-month mortality.^[36]^ Kang et al^[34]^ confirmed that GNRI was associated with poor prognosis in patients with acute ischemic stroke.

#### 3.1.5. Mini nutritional assessment short-form.

Mini nutritional assessment short-form (MNA-SF) is a simple, quick and easy nutritional tool to assess the nutrition status. The MNA-SF includes 6 geriatric-specific assessment questions, which includes BMI, food intake, weight loss, general mobility, acute diseases, psychological stress, and neuropsychological problem.^[37]^ Patients were considered to be of “normal nutritional status” if the MNA-SF screening score value was from 12 to 14, at “risk of malnutrition” with a value between 8 and 12, and “malnourished” with a score of less than 7. Malnutrition is a risk factor for sarcopenia because a decrease in muscle mass reduces the basal metabolic rate in a negative chain reaction that results in lower energy consumption and lower nutrient intake.^[38]^ Tan et al^[39]^ found that nutritional risk/malnourished was significantly associated with 2 to 3 times increased odds of sarcopenia.

#### 3.1.6. Bioelectrical impedance analysis.

Bioelectrical impedance analysis has become quite a popular method for estimating body composition, including muscle mass, considering that it is noninvasive, inexpensive, portable, and easy and quick to use.^[40]^ In recent years, skeletal muscle index (SMI) assessed using bioelectrical impedance analysis have been widely used as indicators of nutritional status and skeletal muscle mass. SMI is included in the international diagnostic criteria for sarcopenia, such as the European Working Group on Sarcopenia in older adults and Asian Working Group for Sarcopenia criteria.^[1,41]^ SMI is calculated as appendicular skeletal muscle mass divided by height squared (kg/m^2^),and the cutoff points for men is SMI < 7.0 kg/m^2^, for women is SMI < 5.7 kg/m^2^.^[1]^ Abe et al^[42]^ confirmed that low PA and SMI was independently and negatively associated with functional recovery in patients with acute stroke.

### 3.2. Prevention and treatment strategy of stroke-related sarcopenia

There is little clinical attention to stroke-related sarcopenia so far. Further, there is no expert consensus on the treatment of stroke-related sarcopenia due to the lack of high-level clinical randomized controlled trial evidence. Given the pathophysiology of stroke-related myasthenia, active nutritional supplement and increased physical activity may play an important role in the prevention and treatment of stroke-related myopathy. Amino acid supplementation can improve the nutritional status of stroke patients, increase muscle mass and prevent muscle loss; vitamin

D combined with whey protein can reduce intramuscular fat infiltration and delay the progression of myasthenia after stroke; high energy intake and avoiding Polypharmacy lifestyle contribute to nutritional intake in stroke patients and reduce the risk of post-stroke malnutrition; exercise can improve muscle mass, mass strength and function, and improve movement ability, and particularly, chair standing exercise can improve stroke-related sarcopenia and the ability of performing activities of daily life.

#### 3.2.1. Amino acids.

The abnormal hyperdecomposition of proteins in tissues caused by metabolic abnormalities after stroke is known as muscle hypercatabolism^[43]^ which results in the breakdown of skeletal and cardiac muscle proteins, releasing amino acids and increasing the body’s energy requirements. The post-stroke period is a critical time to evaluate the metabolic changes associated with hypercatabolism and the role of amino acid supplementation.^[44]^ Although muscle mass is controlled by a complex interaction of many factors, the dynamic balance between protein synthesis and catabolism is the main determinant. It is well-known that protein is essential for many physiological functions, particularly in promoting skeletal muscle growth and maintaining skeletal muscle mass. Significant increases or declines in muscle mass and strength result from sustained changes in muscle protein synthesis, catabolism or a combination of both. Amino acid replacement stimulates protein synthesis by improving aerobic metabolism and increasing insulin-like growth factor levels, which in turn enhance protein synthesis mechanisms.^[43]^ In the early recovery period after stroke, nutritional interventions in the form of amino acid supplementation can improve muscle volume and physical function.^[45]^ Amino acid supplementation can stimulate muscle protein synthesis, improve anabolic activity, restore skeletal muscle function and body function, reduce muscle atrophy, and improve the effectiveness of rehabilitation in stroke patients. A randomized controlled trial by Ikeda et al^[46]^ showed that supplementation with leucine-rich amino acids was effective in improving activities of daily living and grip strength in patients with stroke-associated sarcopenia. Meanwhile, Montenegro et al^[47]^ found that supplementation with branched-chain amino acids had a positive effect on stroke-associated sarcopenia, which indicated that compared with the control group, the skeletal muscle mass index and body function status in the intervention group were significantly improved after supplementing branched-chain amino acid combined with intensive rehabilitation therapy.

#### 3.2.2. Combined supplementation whey protein with vitamin D.

Vitamin D (VD) supplementation is beneficial to the improvement of muscle function in the older adults and is a necessity for muscle maintenance. VD deficiency is a common issue in stroke survivors which is associated with reduced muscle strength, balance and physical performance and low levels of VD are considered a biomarker of mortality and functional prognosis in stroke patients.^[48–50]^ VD can prevent stroke and neurodegeneration through antioxidant mechanisms, regulation of immune response, regulation of calcium homeostasis, inhibition of pro-inflammatory agents and detoxification. Studies^[51]^ have found that VD and VD receptors have an essential role in regulating satellite cell activity, protein synthesis, mitochondrial metabolism, contributing to the maintenance of muscle mass and function. Although there are conflicting findings regarding the effects of VD supplementation on post-stroke rehabilitation, studies^[21]^ suggest that VD has the potential to support neurological function and facilitate the rehabilitation process. A randomized, single-blind controlled trial investigated the effects of whey protein and VD supplementation on muscle mass, muscle decline and clinical outcome in stroke patients in restoration stage.^[52]^ The results found that whey protein and VD supplementation had no positive effect on muscle mass and physical function, but helped to improve BMI, serum triglycerides and fat infiltration into muscle in patients recovering from stroke. Intramuscular fat accumulation, which increases with age and correlates with muscle strength, is a risk factor for restricted activity. Therefore, supplementation with whey protein and VD may inhibit the progression of sarcopenia by reducing intramuscular fat infiltration, maintaining muscle mass in older adult stroke patients and contributing to the long-term prognosis of stroke patients, but more high-quality evidence-based medical evidence is needed.

#### 3.2.3. Changes in diet and lifestyle.

##### 3.1.3.2. High energy intake

Nutritional therapy in stroke patients has been reported to improve activities of daily living by taking high-energy, high-protein supplements,^[53]^ but the efficacy of stroke-related sarcopenia is unknown. Sato et al^[54]^ conducted a study to explore the effect of energy intake and recovery time on the improvement of activities of daily living in patients with stroke-related sarcopenia which included 140 stroke patients (mean age 82.6 years, 67 men). The results showed that adequate energy intake and longer recovery time were strongly associated with improved activities of daily living in patients with stroke-related sarcopenia. Compared with standard diets, High-energy, high-protein oral nutritional supplements may improve functional independence and 6-minute walking distance of the patients with stroke-related sarcopenia.

##### 3.2.3.2. Polypharmacy

Polypharmacy is a common health issue in older adults and is associated with malnutrition and decreased physical function. Studies^[55]^ have shown that overuse of multiple drugs (>10 drugs) in older adults older than 75 years is associated with an increased risk of malnutrition. Therefore, prophylactic polypharmacy may improve sarcopenia, malnutrition, and physical function in the aged. Matsumoto et al^[56]^ conducted the study on the effects of multi-drug combination on nutrition and muscle in older adults after stroke. Of the 361 stroke patients included in the study, 158 were considered to have overused medications, and 57.6% of the over-medicated older patients were diagnosed with sarcopenia. Studies have found that reduced multidrug combination use is associated with improved nutritional intake. In stroke patients with sarcopenia, a decrease in the number of medication during hospitalization was positively correlated with an increase in energy and protein intake. Although, the study did not elaborate on the specific drug reductions, it suggested that reducing the polypharmacy may promote to improve nutritional status in patients with stroke-related sarcopenia. At the same time, high-quality prospective studies on the effects of specific drug reductions and doses on nutritional status are urgently needed to fill the evidence gap.

##### 3.3.2.3. Exercise

Exercise can improve muscle mass, strength and function, improve mobility, and may have a protective and beneficial effect in the prevention and treatment of stroke-related sarcopenia.^[57]^ Therefore, patients with stroke-related sarcopenia may choose to undergo multimodal training in addition to the routine rehabilitation exercise program. Multimodal training includes a combination of resistance training, aerobic training, walking, and balance training.^[58]^ Compared with other exercises, the Chair Stand Exercise is a repetitive, low-intensity, slow, and safe exercise that improves the ability to perform activities of daily living and is effective in increasing muscle mass and strength in stroke and dialysis patients.^[58,59]^ In addition, the exercise does not require any specialized skills, equipment, or facilities, is less costly, and can be performed by individuals or groups of all sizes in a hospital rehabilitation setting.^[52,58]^ Yoshimura et al^[60]^ found that chair standing exercises were positively correlated with improvements in activities of daily living of the patients with stroked-related sarcopenia. In this study, stroke-related sarcopenia patients with systemic resistance exercises such as chair standing exercises on the basis of conventional rehabilitation programs could improve physical function and disease severity of stroke-related sarcopenia. Therefore, in the rehabilitation process of patients with stroke-related sarcopenia, systemic body exercises such as chair standing exercises should be added to the routine rehabilitation program in order to further improve sarcopenia and activities of daily living.

## 4. Conclusions

There is no expert consensus on the management of stroke-related sarcopenia, and nutrition plays a key role in the management of stroke-related sarcopenia. Stroke and sarcopenia are 2 independent but interacting chronic conditions and stroke-associated sarcopenia can be considered as a multifactorial syndrome. Supplementation of essential amino acids, whey protein combined with vitamin D, high-energy diet, avoidance of polypharmacy and increased physical activity level can improve the state of malnutrition in stroke patients, delay the progression of sarcopenia, and improve muscle mass and skeletal muscle index in patients with stroke-related sarcopenia. Current knowledge of the pathogenesis, assessment and prevention of stroke-associated sarcopenia is still limited and there are no drugs available to treat stroke-associated sarcopenia. High quality prospective intervention studies, including nutritional interventions combined with rehabilitative exercise training, are insufficient and strategies for the management of stroke-associated sarcopenia are needed to be further studied in order to improve the quality of life for patients.

## Author contributions

**Conceptualization:** Hongxia Chen.

**Supervision:** Hongxia Chen.

**Writing – original draft:** Zhiqiang Gao.

**Writing – review & editing:** Hongxia Chen.
